# A new method for sequencing the hypervariable *Plasmodium falciparum* gene *var2csa* from clinical samples

**DOI:** 10.1186/s12936-017-1976-8

**Published:** 2017-08-17

**Authors:** Antoine Dara, Mark A. Travassos, Matthew Adams, Sarah Schaffer DeRoo, Elliott F. Drábek, Sonia Agrawal, Miriam K. Laufer, Christopher V. Plowe, Joana C. Silva

**Affiliations:** 1 Division of Malaria Research, Institute for Global Health, University of Maryland School of Medicine, Baltimore, MD USA; 2 Institute for Genome Sciences, University of Maryland School of Medicine, Baltimore, MD USA; 3 Department of Microbiology and Immunology, University of Maryland School of Medicine, Baltimore, MD USA

**Keywords:** *var2csa*, Sequencing, PacBio, Malaria, Vaccines

## Abstract

**Background:**

VAR2CSA, a member of the *Plasmodium falciparum* erythrocyte membrane protein 1 (PfEMP1) family, mediates the binding of *P. falciparum*-infected erythrocytes to chondroitin sulfate A, a surface-associated molecule expressed in placental cells, and plays a central role in the pathogenesis of placental malaria. VAR2CSA is a target of naturally acquired immunity and, as such, is a leading vaccine candidate against placental malaria. This protein is very polymorphic and technically challenging to sequence. Published *var2csa* sequences, mostly limited to specific domains, have been generated through the sequencing of cloned PCR amplicons using capillary electrophoresis, a method that is both time consuming and costly, and that performs poorly when applied to clinical samples that are commonly polyclonal. A next-generation sequencing platform, Pacific Biosciences (PacBio), offers an alternative approach to overcome these issues.

**Methods:**

PCR primers were designed that target a 5 kb segment in the 5′ end of *var2csa* and the resulting amplicons were sequenced using PacBio sequencing. The primers were optimized using two laboratory strains and were validated on DNA from 43 clinical samples, extracted from dried blood spots on filter paper or from cryopreserved *P. falciparum*-infected erythrocytes. Sequence reads were assembled using the SMRT-analysis ConsensusTools module.

**Results:**

Here, a PacBio sequencing-based approach for recovering a segment encoding the majority of VAR2CSA’s extracellular region is described; this segment includes the totality of the first four domains in the 5′ end of *var2csa* (~5 kb), from clinical malaria samples. The feasibility of the method is demonstrated, showing a high success rate from cryopreserved samples and more limited success from dried blood spots stored at room temperature, and characterized the genetic variation of the *var2csa* locus.

**Conclusions:**

This method will facilitate a detailed analysis of *var2csa* genetic variation and can be adapted to sequence other hypervariable *P. falciparum* genes.

**Electronic supplementary material:**

The online version of this article (doi:10.1186/s12936-017-1976-8) contains supplementary material, which is available to authorized users.

## Background

Placental malaria (PM) is characterized by the massive accumulation of *Plasmodium falciparum*-infected erythrocytes in the placental intervillous space. The sequestration of infected erythrocytes in host tissue, such as the placenta, is mediated by adhesin proteins in the *P. falciparum* erythrocyte membrane protein 1 (PfEMP1) family, which are encoded by the highly diverse *var* gene family [1]. PfEMP1s are a complex family of proteins ranging from 300 to 350 kDa that are composed of two to nine Duffy binding-like (DBL) domains and one to two cysteine-rich interdomain regions (CIDR) [2, 3]. Parasites sequester in many different organs, including the brain, heart, lungs, liver, and placenta (reviewed in [4]). *Plasmodium falciparum*-infected erythrocytes collected from placentas display a unique cyto-adherence phenotype [5], and they bind to glycosaminoglycan chondroitin-4-sulfate (CSA) expressed in the placenta, but not to endothelial receptors such as CD36 or ICAM-1 [6, 7]. The ligand mediating this interaction with CSA is a PfEMP1 protein known as VAR2CSA [8]. VAR2CSA is a target of naturally acquired immunity [9–12], making it an attractive vaccine candidate against placental malaria.

**Fig. 1:**
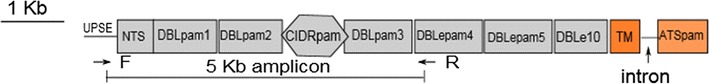
Schematic representation of the locus encoding VAR2CSA. Target-specific primers are shown, with the arrows indicate forward (F) and reverse (R) primers targets. *UPSE* upstream promoter sequence, *NTS* N-terminal sequence, *DBL* Duffy-like binding domain, *CIDR* cysteine-rich inter-domain, *TM* transmembrane, *ATS* acidic terminal sequence, *pam* pregnancy-associated malaria. The intercellular region is colored in *grey*, and the TM and ATS are in *orange*

Optimizing the design of a vaccine based on VAR2CSA is challenging given VAR2CSA’s size and high sequence diversity, particularly given that allele specificity has been a concern for malaria vaccines based on other *P. falciparum* antigens, such as the apical membrane antigen 1, AMA-1 [13, 14] or the circumsporozoite protein, CPS [15]. VAR2CSA is a multi-domain protein of 340 kDa with an extracellular region comprised of six DBL domains and one CIDR domain, a transmembrane domain and an intracellular region primarily consisting of an acidic terminal sequence (Fig. 1). Each domain contains between 300 and 500 amino acids. In vitro studies have shown that several domains bind to CSA [16], but the minimal CSA-binding region, known as ID1-ID2a, has been mapped to the second DBL domain (DBLpam2) flanked by interdomain 1 and the first 93 amino acids of the CIDRpam domain [17]. This region is currently a target for VAR2CSA-based vaccine development, even though other studies have suggested that other domains are important for naturally acquired antibodies [9, 11, 18–24].

Sequencing *var2csa* from clinical isolates can provide a framework for VAR2CSA-based vaccine development, but *var2csa’*s genetic complexity and large size have limited its successful sequencing from field samples. Capillary sequencing, also known as Sanger sequencing, has been used to sequence a limited number of full-length *var2csa* genes from clinical isolates [2, 25]. Most sequences have been generated using Sanger sequencing and span only single, specific *var2csa* domains [25–27], even though there is no consensus on which domain(s) is important for a sub-unit vaccine. This method requires a priori knowledge of the sequence of primer-binding regions, limiting the number of variants that can be obtained and analysed. It is also time consuming, as it requires cloning of PCR products prior to sequencing. In the case of polyclonal infections, which are common in clinical isolates, this method is even less efficient and may lead to the generation of chimeric sequences. Ilumina next-generation sequencing has been used to generate whole genome sequence data of *P. falciparum*. However, the difficulty of assembling *var* genes from short read next-generation whole genome sequence data, such as those generated with Illumina sequencing technologies, has resulted in limited success in generating full-length, or even large fragments of, *var2csa*. Therefore, alternative ways to sequence full-length *var2csa*, or a large critical fragment (spanning several domains) of *var2csa*, from clinical specimens are needed.

A next-generation sequencing platform from Pacific Biosciences (PacBio) has the potential to generate complete *var2csa* sequences due to its ability to generate long reads. PacBio sequencing is also appealing because it is high-throughput, as samples can be multiplexed. More importantly, the assembly does not require a reference sequence making it suitable for a highly polymorphic gene such as *var2csa*. Targeted PacBio sequencing has been used to sequence complex loci in humans [28], as well as in *P. falciparum* [29]. This approach was modified here to develop a new sequencing assay for *var2csa*, which combines long-range PCR with PacBio sequencing and assembly. This amplicon sequencing of the *var2csa* N-terminal region captures approximately half of the full-length *var2csa*, including ID1-ID2a, the primary focus of the current VAR2CSA-based phase I vaccine trial. The results of this novel assay are reported, together with an evaluation of the genetic diversity of this segment of VAR2CSA. The methods described can be adapted to other hypervariable genes or gene families.

## Methods

### Sites and samples

As part of International Centers of Excellence for Malaria Research (ICEMR) and malaria-in-pregnancy (MIP) studies, samples were collected from children, men and women in Ndirande, a peri-urban township of Blantyre, in Malawi, where malaria transmission occurs throughout the year with a seasonal peak during a rainy season from December to March. Forty-three specimens, including 19 blood-spotted Whatman 3MM filter papers that had been stored at room temperature and 24 cryopreserved parasites, were randomly selected. Genomic DNA was extracted using a Qiagen Midi Kit according to manufacturer’s instructions. Four positive controls were used: 3D7, HB3, and synthetic mixtures of 70% 3D7 plus 30% HB3 and vice versa. 3D7 and HB3 each had an initial concentration of 1 ng/µl.

### Primer design and sequencing template preparation

Primers that flank the region spanning the *var2csa* upstream promoter (position 500 bp upstream of start codon) to the DBLepam4 domain were designed using 20 aligned *var2csa* sequences from public databases (GenBank and VarDom) along with 12 *var2csa* upstream promoter (UPSE) sequences obtained previously [30]. Each secondary PCR primer was labelled using PacBio barcode sequences (listed in Additional file 1: Table S1) to identify sequences from individual samples. High-fidelity Takara LA Taq^®^ Polymerase (TAKARA BIO INC, Shiga, Japan) was used for the PCR with conditions listed in Additional file 1: Tables S2 and S3. An expected secondary PCR product of 5364 bp was resolved on 0.8% agarose gel and visualized using Gel Doc XR+ System (Bio-Rad, Hercules, CA, USA). Successfully amplified samples were purified using a MultiScreen filter plate (MultiScreen, Tullagreen, Germany). DNA concentration was measured using a Quant-iT PicoGreen dsDNA assay (Life Technologies, Carlsbad, CA, USA) according to manufacturer’s instructions. Subsequently, 48 equimolar barcoded amplicons were pooled to a final total of 2 µg of DNA.

### Sequencing

Purified, pooled amplicons were sequenced using Single Molecule Real Time (SMRT) technology on a PacBio RS II sequencer (Menlo Park, Pacific Biosciences, California, USA), at the Genomics Resource Center of the Institute for Genome Sciences, University of Maryland School of Medicine, USA. Briefly, libraries were constructed by ligating SMRTbellTM adapters to the barcoded-pooled amplicon template. One SMRT™ cell with P4-C2 chemistry (P4 polymerase with C2 sequencing chemistry) was used. A 180-min movie was performed on PacBio RS II to generate the reads.

### Data processing

The secondary analysis was performed with the PacBio SMRT Analysis v2.3.0 package, using the long amplicon analysis (LAA) algorithm with the following options—minLength 3250—minSnr 4. This algorithm enables read clustering, phasing and consensus calling, and consensus filtering. The pooled amplicon data were de-multiplexed by barcode. The predicted accuracy of 95%, which is defined as the threshold below which a haplotype (consensus sequence) is considered as noise, was used to filter high-quality consensus sequences for downstream analysis.

To determine the sequence accuracy of the positive controls, amplicon consensus sequences were aligned to coding sequences of the respective strains from which the amplicons were generated. The corresponding test coding sequences were extracted and the phylogenetic tree was built using the Maximum Likelihood method implemented in MEGA 6.0 [31].

Sequences from the same sample that shared 99% identity at the nucleotide level were clustered using CD-HIT [32]. This conservative threshold was chosen assuming that the residual sequencing error was 1% based on data from the resequenced positive controls. Sequences with depth of coverage equal to or greater than 100× were considered for downstream analysis. This 100× threshold was chosen because this was the minimum depth of coverage at which a perfect consensus sequence of the reference 3D7 was obtained. Then the amplicon sequences were fed into OrfPredictor [33] to predict open reading frames, and corresponding amino acid sequences were generated. Amino acid sequences were used to annotate constituent domains using the VarDom 1.0 server [2]. Multiple alignments were performed using MAFFT with L-INS-i accuracy-oriented options. The region in the multiple alignments corresponding to the FCR3 variant of ID1-ID2a was extracted (1158–3075 corresponding to amino acid position N386 to D1025).

For the genetic diversity analysis, sequences corresponding to the coding regions were extracted from the full-length amplicon. Likely frameshift errors were corrected with AlignWise [34]. Sequences were then aligned with MAFFT with the L-iNSi option. DnaSP v5 [35] was used to compute population genetic parameters, including nucleotide diversity, which is the average number of nucleotide differences per site between any two given sequences; haplotype diversity, which is the probability that two randomly sampled alleles are different; and Tajima’s D neutrality test, which is used to infer the selective forces acting on a locus based on the difference between the number of segregating sites and the average number of nucleotide differences.

## Results

### Robustness of the amplification protocol on clinical samples

The amplification protocol was developed using genomic DNA from two laboratory parasite reference strains, 3D7 and HB3. The primers are designed to bind to the VAR2CSA-specific upstream promoter, UPSE, and to the DBLepam4 domain, to amplify a segment close to 5 kb in length (Fig. 1). This primer combination readily amplified *var2csa* from both of these lines. To test the robustness of the protocol, it was applied to clinical samples including DNA extracted from blood-spotted filter papers and from cryopreserved specimens obtained from 81 clinical malaria samples from Malawi. A long PCR amplicon of the expected size was obtained from both of these sample types. The PCR was successful for all cryopreserved specimens (24/24), whereas only 33% (19/57) of the tested filter samples yielded visible PCR products by gel electrophoresis. Therefore, the starting combined pool consisted of 43 amplicons, in addition to the four positive controls from 3D7 and HB3 (described in “Methods” section).

### PacBio-generated long sequence reads

**Fig. 2:**
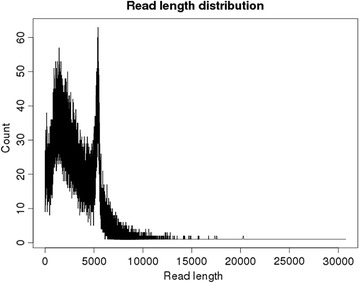
Distribution of read length. A 5.3 kb-long insert library was sequenced at 180 min. The raw reads were filtered and split based on adapters to generate sub-reads referred to here as ‘reads’. The length of each read is plotted against the read counts. The first peak corresponds to reads with length around 2.7 kb and the second peak corresponds to reads that spanned the full-length 5 kb amplicon. Most of the reads were shorter than the amplicon size, but there was a substantial number of long reads that spanned the full-length amplicon. With the very long reads (>5 kb), the adapters delimiting the subreads were either missing or not identified

All 43 amplicons and the 4 controls (see “Methods”) were multiplexed in one PacBio SMRT cell, yielding 95,220 raw polymerase reads, with average and median length of 5.3 and 4.4 kb, respectively (Additional file 2: Figure S1). The raw reads were processed by removing the circularization (barbell) adaptors, resulting in 161,534 sub-reads. Applying a filter with quality score cut-off of 0.75 (Phred score ≥20) and read length ≥50 bp, reduced this set to 143,603 sub-reads, with length ranging from 50 to 30,000 base pairs, for an average of 3055 sub-reads per sample. The presence of sub-reads longer than 5 kb indicates that some of the reads contain missing or unidentified barbell adaptors. The sub-read length distribution was bimodal with one peak around 2 kb and a second peak at the expected amplicon size of approximately 5 kb, and a median length of 2728 bp (Fig. 2).

### Validation of *var2csa* reconstruction protocol

To validate this amplification, sequencing and assembly approach, the nucleotide sequence of the positive controls was compared to the published VAR2CSA sequences for each strain (PFL0030c, HB3*var2csa*A, and HB3*var2csa*B). The sequences recovered from positive controls were virtually identical to the respective reference *var2csa* sequences in the 3D7 and HB3 genomes, with nucleotide sequence identity from 99.8 to 100% between the re-sequenced consensus and the corresponding reference allelic sequences (Table 1).

**Table 1:** Comparison between reconstructed PacBio sequences and the respective reference *var2csa* alleles in 3D7 and HB3

Reference *var2csa* gene	Re-sequenced controls^a^	Coverage depth	Sequence identity (%)	Reference length^b^	PacBio length^c^	Deletion length (bp)
PFL0030c	3D7_pacbio100_NumReads445	445	100	4843	4843	0
PFL0030c	3D7_pacbio70_NumReads119	119	99.94	4843	4840	3
PFL0030c	3D7_pacbio30_NumReads82	82	99.96	4843	4841	2
HB3*var2csa*A	HB3_pacbio100_NumReads101	101	99.98	4981	4980	1
HB3*var2csa*B	HB3_pacbio_100_NumReads100	100	99.98	4939	4938	1
HB3*var2csa*A	HB3_pacbio70_NumReads46	46	99.82	4981	4972	9
HB3*var2csa*B	HB3_pacbio70_NumReads59	59	99.94	4939	4936	3
HB3*var2csa*A	HB3_pacbio30_NumReads167	167	99.98	4981	4980	1
HB3*var2csa*B	HB3_pacbio30_NumReads70	70	99.94	4939	4936	3

^a^The type of control is indicated by the name of the consensus sequence: pacbio100: amplicon obtained from a single reference strain; pacbio30: amplicon obtained from a mix of reference strains in which the named strain was present at 30% of the DNA sample; pacbio70: similarly defined as pacbio30, with the named strain representing 70% of the DNA sample

^b^Length of the reference *var2csa* allele

^c^Length of the reconstructed *var2csa* coding sequence

**Fig. 3:**
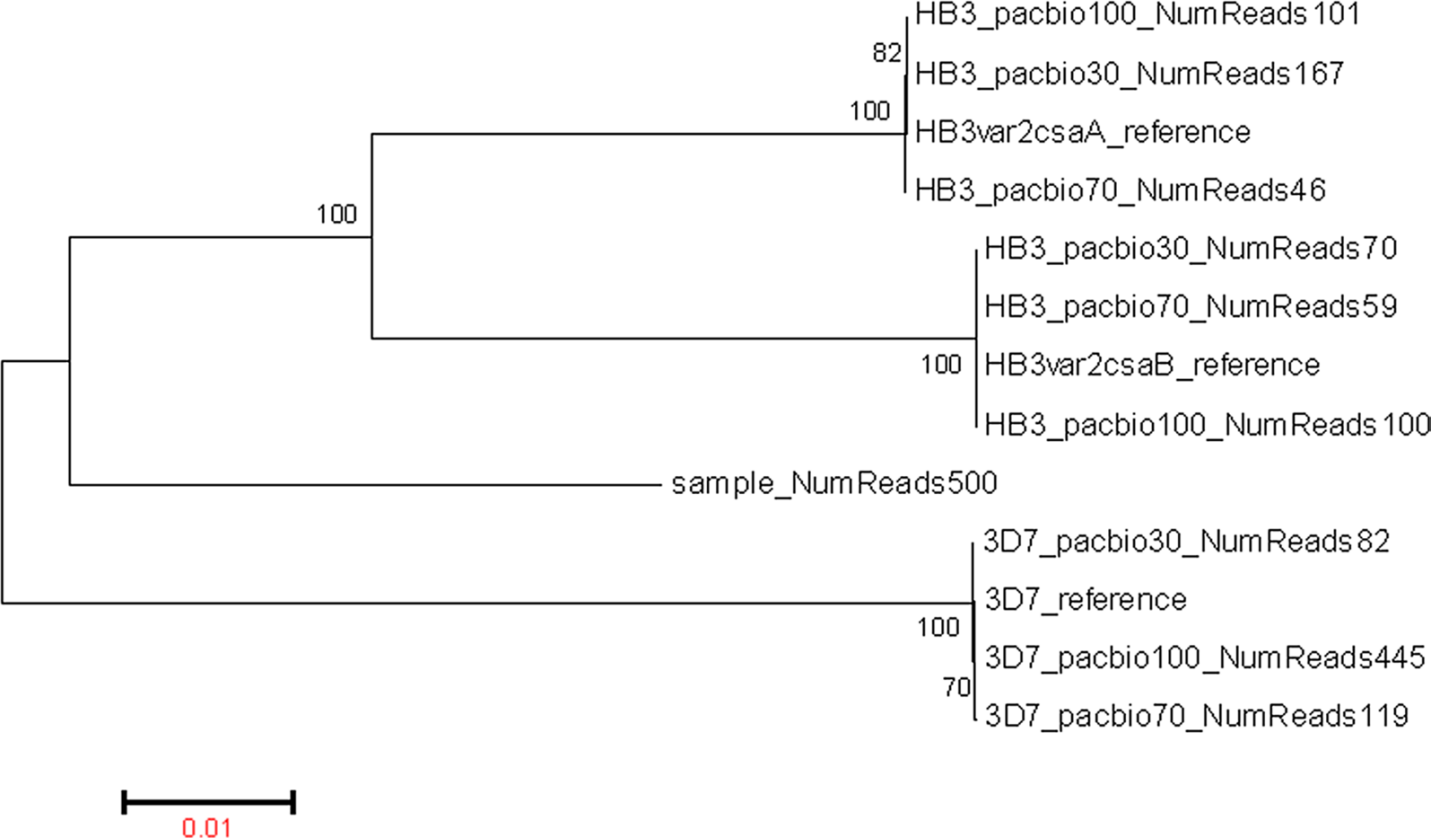
A maximum likelihood phylogenetic tree of reference and PacBio-reconstructed *var2csa* allelic sequences. Each sequence is named using the strain’s name (e.g. 3D7) followed by the sequencing platform (PacBio), with the proportion of the DNA composed of the specific strain (30, 70, 100%) and the number of reads used to form the consensus sequences (NumReadsxx). A phylogenetic tree of the reconstructed alleles was built using maximum likelihood, as implemented in MEGA v.6 with default parameters. The analysis showed PacBio-generated sequences to cluster with reference alleles as expected, and detected minor alleles. 3D7_PacBio denotes PacBio-sequenced 3D7, HB3_PacBio PacBio-sequenced HB3, 3D7_70 a 3D7 sequence resolved from a mixture of 70% 3D7 and 30% HB3, and HB3_70 an HB3 allele from a mixture of 30% 3D7 and 70% HB3. Of note, HB3 has two alleles of *var2csa* (A, B); therefore, a mixture of 3D7 and HB3 are expected to have 3 alleles. The second allele of HB3, representing 15% of the mixture in theory, was not detected in the 70% 3D7 and 30% HB3 mixture. Bootstrap support values are indicated. The *scale* reflects sequence divergence per base pair. 3D7_ref, HB3*var2csa*A, and HB3*var2csa*B are the allelic sequences obtained from the VarDom database

To evaluate the method’s performance with polyclonal infections, a synthetic mixture containing *var2csa* sequences from the two reference strains was sequenced. Each allele from the mixture was successfully reconstructed (Fig. 3). Sequences recovered from HB3 alone and from synthetic mixtures had between 99.82 and 99.98% identity to HB3 reference sequences (Table 1). The lack of complete sequence identity was related to low depth of coverage. The non-mixed 3D7 (referred to as 3D7_pacbio100 in Table 1) had 445-fold coverage, whereas the HB3 and the mixed-controls have a coverage range between 46- and 167-fold. The only difference observed between reconstructed and the reference sequence were deletions that occurred at homopolymeric regions (Additional file 1: Table S4).

To determine the minimum number of reads required for an accurate assembly, reads were subsampled to simulate the 3D7 assembly at different coverage levels. At 100-fold coverage, a sequence identical to the reference was obtained. However, when a larger number of reads was used, some artifacts were observed in the assemblies, such as one deletion at 125-fold and two deletions at 150-fold coverage, suggesting that as read coverage increases so does the likelihood of repeated erroneous sequences. Therefore, the relationship between accuracy and coverage was not always linear (Table 2).

**Table 2:** Simulated 3D7 amplicon assembly at different coverage levels

Coverage level	Number of INDELs	Sequence identity (%)
50×	4	99.91
75×	3	99.94
100×	0	100
125×	1	99.98
150×	2	99.96
200×	1	99.98
250×	0	100
300×	1	99.98
445×	0	100

### Application of the sequencing method to clinical samples

The parameter settings used for sub-read assembly, optimized during the reconstruction of the reference 3D7 allele, were then applied to the clinical samples. After filtering, read clustering, and chimeric sequence removal, 135 consensus sequences were generated from the 43 samples. After collapsing nearly identical sequences (see “Methods”) from the same sample using a threshold for minimum nucleotide sequence identity of 99%, which is a cut-off based on the re-sequenced positive control (Table 1), the number of sequences was reduced to 99. A subsequent filter, using a minimum coverage threshold of 100-fold, yielded 51 sequences from 40 samples, with a mean coverage of 280-fold (100–500) (Additional file 2: Figure S2). All samples but one yielded one or more unique amplicon sequences (Additional file 3).

To further evaluate the specificity of the new protocol, in particularly whether in fact *var2csa* sequences were amplified, the translated sequences were annotated using the VarDom 1.0 server [2], which uses a Hidden Markov Model to identify PfEMP1 protein domains. *var2csa*-specific homology blocks (HB) and domains (DBLpam1, DBLpam2, CIDRpam, and DBLpam3) were detected in all samples with a single, intact open reading frame (30 sequences with length ≥1400 amino acids). The remaining 21 amplicons were also *var2csa* sequences, validated as such using blast searches against NCBI’s non-redundant sequence database, with frameshift mutations that resulted in in-frame stop codons. These results validate our approach to generating *var2csa* sequences (Additional file 2: Figure S3).

### Genetic diversity of *var2csa*

**Fig. 4:**
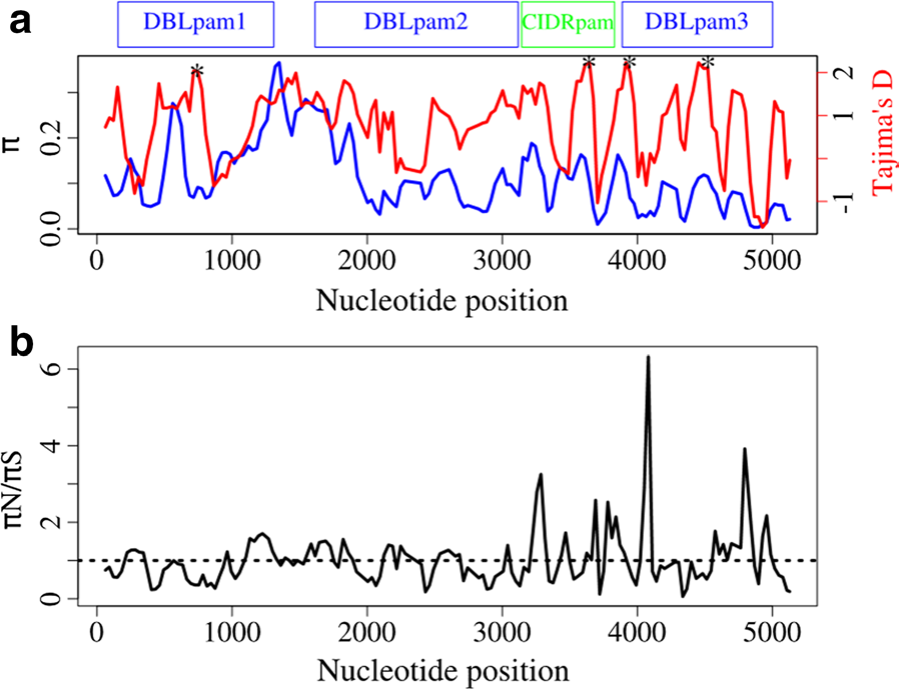
Distribution of genetic diversity in the 5′ region of *var2csa*. **a** Distribution of nucleotide diversity and Tajima’s D across *var2csa*. Sliding window analyses of the nucleotide nucleotide diversity (*blue*) and Tajima’s D values (*red*) are plotted against the length of the coding region, with a window size of 99 nucleotides and step size of 30 nucleotides. The highest peak of nucleotide diversity corresponds to the inter domain 1 region. An *asterisk* denotes a position that significantly deviated from neutrality (p < 0.05), which included positions 666–764, 3563–3694, 3866–3964, and 4391–4573 in the alignment. The VAR2CSA segment analysed in this study is at the *top of the plot*. **b** Sliding window analysis of non-synonymous to synonymous mutation rate is plotted (*π*_N_/*π*_S_ ratio) against the length of the coding region, with a window size of 99 nucleotides and step size of 30 nucleotides. The *blue dashed line* denotes a *π*_N_/*π*_S_ ratio of 1; *π*_N_/*π*_S_ >1 indicates an excess of non-synonymous mutations, whereas a ratio <1 is indicative of an excess of synonymous mutations. The VAR2CSA segment analysed in this study is shown *above the plot*

To characterize the genetic variation in the 5′ region of the locus, the 5 kb segment amplified from the *var2csa* gene was used. The analyses were based only on sequences with an intact open reading frame that spanned the whole 5 kb region; partial sequences were excluded. A total of 30 full-length sequences were included in the analysis. A total of 4613 nucleotide sites (sites with gaps/missing data were excluded) were analysed, of which 1408 were polymorphic. The overall nucleotide diversity across this region was 11.16% (*π* = 0.1116). When analysed by constituent domains, DBLpam3 showed the least variation, with a nucleotide diversity of *π* = 0.056, followed by CIDRpam (*π* = 0.104) and DBLpam2 (*π* = 0.106), whereas DBLpam1 was the most diverse domain, with *π* = 0.138 (Table 3). To further determine the distribution of genetic variation across this region of the gene, we calculated nucleotide diversity using a sliding window of 99 nucleotides in length, and sliding step of 30 nucleotides, across the coding region of the 5 kb segment. The most polymorphic regions corresponded to the interdomain region between DBLpam1 and DBLpam2, as well as a central region of DBLpam1 and the N-terminal region of DBLpam2 (Fig. 4a).

**Table 3:** Genetic variation across the whole segment and in individual domains

Domain	Region	n	Haplotype diversity (Hd)	Nucleotide diversity (π)	Tajima’s D	Tajima’s D p value
DBLpam1	153–1308	30	0.998	0.1389	0.6013	>0.1
DBLpam2	1614–3117	30	0.998	0.10627	1.0243	>0.1
CIDRpam	3144–3831	30	0.998	0.1048	0.9163	>0.1
DBLpam3	3888–5001	30	0.998	0.05558	0.9847	>0.1
ID1-ID2a	1185–3297	30	0.998	0.1467	1.2457	>0.1
Total	1–5169	30	0.998	0.11168	0.9425	>0.1

To better understand the evolutionary forces that shape the genetic variation of *var2csa*, we estimated the distribution of two different statistics across the this segment, namely the ratio *π*_N_/*π*_S_, which measures the ratio of non-synonymous to synonymous mutations per site, with expected value of zero when the types of mutations are randomly distributed; and Tajima’s D statistic, which assesses the evolutionary forces operating on a locus, with expected value of zero under neutral evolution. A *π*_N_/*π*_S_ ratio greater than 1 is suggestive of positive selection, whereas a ratio of less than 1 suggests purifying selection. On antigenic loci, positive values of Tajima’s D are suggestive of balancing selection, while negative values are likely due to purifying selection. None of the statistics showed a statistically significant departure from neutrality either across the whole 5 kb segment or on individual domains (Table 3). The overall ratio *π*_N_/*π*_S_ was 0.89, indicating a slight excess of synonymous over non-synonymous polymorphism.

To determine whether specific short regions of the gene show evidence of positive selection, these two statistics were estimated using a sliding window analysis, with a window size of 99 nucleotides and a sliding step size of 30 nucleotides (Fig. 4). The Tajima’s D analysis identified some regions where the distribution of genetic variation departed from neutrality (p < 0.05), corresponding to positions 666–764 in the DBLpam1 domain; 3563–3694 in the CIDRpam domain; and 3866–3964 and 4391–4573 in the DBLpam3 domain (Fig. 4a). One striking *π*_N_/*π*_S_ peak was present, which mapped to DBLpam3 (alignment positions 4041–4139 with πN/πS ratio of 6.47) but with relatively low overall *π*, suggesting that whatever polymorphism there is in this region is non-synonymous. Two intermediate peaks were also observed, which mapped to the C-terminal regions of the CIDRpam domain (alignment positions 3246–3344 with πN/πS of 2.73) and the DBLpam3 domain (4707–4856 with πN/πS ratio of 3.92), each with a significantly high value of Tajima’s D, suggesting that balancing selection is acting at these regions (Fig. 4). Taken together, these results showed that the N-terminal of *var2csa* is highly polymorphic. To put *var2csa* genetic diversity in context, its nucleotide diversity is ten times higher than that in the *P. falciparum* apical membrane antigen 1 (AMA1), which itself is one of the most diverse loci in *P. falciparum* [14]. The high values of Tajima’s D in the DBLpam1, the CIDRpam and the DBLpam3 domains of the protein are consistent with balancing selection. These findings are in line with those from other studies that found that variable blocks in DBLpam1, DBLpam2 and DBLpam3 are evolving under positive selection [26, 36, 37]. Taken together, these analyses suggest that host immune pressure drives the diversity in these segments, likely under a selective regimen of frequency-dependent selection that favours rare alleles.

### The minimal CSA-binding region, a current vaccine target, is highly diverse

Sequence diversity of the CSA binding region, located between coordinates 1185–3297 in Fig. 4, was characterized since this region is a target of the current VAR2CSA sub-unit vaccine [17]. The overall amino acid sequence identity was 74% and the average nucleotide diversity was 14.6% (Table 3), with a proportion of segregating sites of 53%. The nucleotide diversity in this region is higher than any of the values calculated for individual VAR2CSA domains.

## Discussion

A better understanding of the genetic variation of VAR2CSA is critical for development of a broadly effective vaccine against placental malaria. However, characterization of its encoding gene from clinical samples has had limited success due to difficulties in amplifying and assembling this hypervariable member of a multigene family from malaria infections, with issues compounded in polyclonal infections. A novel sequencing approach was developed using long range PCR combined with long PacBio sequencing of the resulting amplicon spanning ~5 kb of the extracellular region of *var2csa*. This strategy obviated the need for cloning and allowed us to generate sequences of large fragments of *var2csa*, capturing the majority of the sequence encoding the extracellular region of the *var2csa* locus. This approach was validated using published sequences from laboratory parasite lines. As proof-of-concept, this approach was applied to two types of field samples, albeit with different success rate.

In this study, two well-characterized laboratory strains were used to optimize the amplification and assembly protocols. 3D7 has one allele of *var2csa*, whereas HB3 harbors two alleles. The PacBio re-sequenced 3D7 *var2csa* allele, assembled from reads with high depth of coverage, is identical to the reference strain. On the other hand, the HB3 *var2csa* reconstructed alleles harbored some residual sequencing errors. All of the errors observed, which were associated with low coverage, were deletions in homopolymeric regions. These findings are in line with those from a previous study that used PacBio targeted sequencing for bacterial 16S rRNA [38] and 454 pyrosequencing amplicon sequencing in *Plasmodium* [39].

Blood-spotted dried filter papers are widely used for molecular assays in epidemiological studies, which can be easily collected and stored during clinical studies. Promisingly, a 5 kb fragment from DNA isolated from such filter papers was often amplified and sequenced, even though the amplification success rate was lower than that of DNA obtained from cryopreserved samples. The limited success of the primers on filter papers could be due to degraded and/or limited amounts of DNA. Further efforts to optimize the DNA extraction and amplification methods from dried blood spot samples are ongoing. Meanwhile, current results suggest that, for any studies that rely on the generation of long PCR amplicons from DNA extracted from filter paper, this type of sample should be kept frozen, or at minimum refrigerated, in order to prevent DNA degradation.

In field settings, multiple infections are common and may undermine the ability of the traditional Sanger platform to sequence clinical specimens directly, without cloning. To examine whether polyclonality poses a challenge for a combined approach of PCR amplification and PacBio sequencing, we artificially created a mixture of laboratory strains. This approach successfully reconstructed alleles present at lower frequency (~23%) in the mixture. However, these minor alleles had lower read coverage and, therefore, slightly more errors. Therefore, current results suggest that in order to obtain highly accurate allelic sequences, additional sequencing may be required when initial consensus sequences are inferred from low coverage levels. However, as mentioned above, all errors observed were deletions in homopolymer stretches. This new method offers a clear advantage over traditional cloning approaches, as it allowed generation of long reads, highly accurate sequences, and was suitable for clinical samples. More importantly, as the protocol uses de novo assembly of full-length amplicons to reconstruct *var2csa*, it allows the accurate reconstruction of *var2csa* sequences from polyclonal clinical specimens.

While this method shows a potential value of PacBio sequencing of amplicons for complex genes, this approach has some limitations. The results reported here were generated from only one SMRT cell, thus limiting depth of coverage and potentially precluding obtaining high-quality sequences for minor alleles, unless funding is available for additional sequencing. Although PacBio sequencing has some residual noise at low depth of coverage, it is anticipated that increasing the number of SMRT cells or decreasing the number of samples to multiplex, and the use of the upgraded P6-C4 chemistry, will improve the outcome of this approach. The sequencing protocol presented here could serve as a potential tool to study *var2csa*. The approach will serve as a framework to characterize the extent of the genetic diversity in different regions of VAR2CSA, information that can then be used to inform rational vaccine design. For instance, this protocol can be used to identify the most prevalent variants to be included in a vaccine that takes into consideration sequence diversity or to identify variants or motifs that are likely to have tropism for receptors overrepresented in placental tissue.

## Conclusions

A new high-throughput method has been developed that uses a combination of long amplicon generation and single molecule sequencing for the locus encoding VAR2CSA. Results show that this method is robust in sequencing *var2csa*, and that the VAR2CSA N-terminal region, including the receptor-binding region, is highly diverse in Malawian samples. The method described here can be an effective approach to study highly polymorphic and complex malaria antigens that have historically been challenging to sequence with traditional platforms.

## Additional files

**Additional file 1:** Supplemental tables.

**Additional file 2:** Supplemental figures.

**Additional file 3:** Nucleotide sequences for the var2csa alleles amplified, in fasta format.
