# Supplementary material for: A new method for sequencing the hypervariable Plasmodium falciparum gene var2csa from clinical samples
Source: Malar J. 2017 Aug 17;16:343. doi: 10.1186/s12936-017-1976-8 (PMC5561619; doi:10.1186/s12936-017-1976-8)
Supplement: Supplementary file 1 — Additional file 1: Supplemental tables. [file 12936_2017_1976_MOESM1_ESM.docx]

**A new method for sequencing the hypervariable *Plasmodium falciparum* gene *var2csa* from clinical samples.**

Antoine Dara, Mark A. Travassos, Matthew Adams, Sarah Schaffer DeRoo, Elliott F. Drábek, Sonia Agrawal, Miriam K. Laufer, Christopher V. Plowe, Joana C. Silva

**Additional file 1**

Table S1: Barcoded primers

| Primer ID | Sequence |
| --- | --- |
| Frag1_InF_BC5 | GGTAGACGTACGCTCGTCATAGTGATGTATGTGTTTATGGAATAACTAGC |
| Frag1_InF_BC6 | GGTAGTGTGAGTCAGTACGCGGTGATGTATGTGTTTATGGAATAACTAGC |
| Frag1_InF_BC7 | GGTAGAGAGACACGATACTCAGTGATGTATGTGTTTATGGAATAACTAGC |
| Frag1_InF_BC8 | GGTAGCTGCTAGAGTCTACAGGTGATGTATGTGTTTATGGAATAACTAGC |
| Frag1_InF_BC9 | GGTAGAGCACTCGCGTCAGTGGTGATGTATGTGTTTATGGAATAACTAGC |
| Frag1_InF_BC10 | GGTAGTCATGCACGTCTCGCTGTGATGTATGTGTTTATGGAATAACTAGC |
| Frag1_InF_BC11 | GGTAGAGAGCATCTCTGTACTGTGATGTATGTGTTTATGGAATAACTAGC |
| Frag1_InF_BC12 | GGTAGCGCATCGACTACGCTAGTGATGTATGTGTTTATGGAATAACTAGC |
| Frag1_InF_BC13 | GGTAGCGTAGCGTGCTATCACGTGATGTATGTGTTTATGGAATAACTAGC |
| Frag1_InF_BC14 | GGTAGATGCTGATGACTGCGAGTGATGTATGTGTTTATGGAATAACTAGC |
| Frag1_InF_BC15 | GGTAGTGCGTGAGCTGTACATGTGATGTATGTGTTTATGGAATAACTAGC |
| Frag1_InF_BC16 | GGTAGCGATCATCTATAGACAGTGATGTATGTGTTTATGGAATAACTAGC |
| Frag1_InF_BC17 | GGTAGCGACGTATCTGACAGTGTGATGTATGTGTTTATGGAATAACTAGC |
| Frag1_InF_BC18 | GGTAGCACGTCACTAGAGCGAGTGATGTATGTGTTTATGGAATAACTAGC |
| Frag1_InF_BC19 | GGTAGTGTCGCAGCTACTAGTGTGATGTATGTGTTTATGGAATAACTAGC |
| Frag1_InF_BC20 | GGTAGCATACGCTGTGTAGCAGTGATGTATGTGTTTATGGAATAACTAGC |
| Frag1_InF_BC21 | GGTAGAGTCGCATGACTGTGTGTGATGTATGTGTTTATGGAATAACTAGC |
| Frag1_InF_BC22 | GGTAGCAGTACTGCACGATCGGTGATGTATGTGTTTATGGAATAACTAGC |
| Frag1_InF_BC23 | GGTAGGTGCTGAGCATCAGACGTGATGTATGTGTTTATGGAATAACTAGC |
| Frag1_InF_BC24 | GGTAGCACTGATCGATATGCAGTGATGTATGTGTTTATGGAATAACTAGC |
| Frag1_InF_BC25 | GGTAGTACAGTGTCTGCTGCGGTGATGTATGTGTTTATGGAATAACTAGC |
| Frag1_InF_BC26 | GGTAGTACAGATAGTGTAGCGGTGATGTATGTGTTTATGGAATAACTAGC |
| Frag1_InF_BC27 | GGTAGTCGTAGAGCTCGAGACGTGATGTATGTGTTTATGGAATAACTAGC |
| Frag1_InF_BC28 | GGTAGGAGCTGCGCACTCGATGTGATGTATGTGTTTATGGAATAACTAGC |
| Frag1_InF_BC29 | GGTAGGCGATGTCGCTATGTGGTGATGTATGTGTTTATGGAATAACTAGC |
| Frag1_InF_BC30 | GGTAGCGAGAGTCAGCGCATAGTGATGTATGTGTTTATGGAATAACTAGC |
| Frag1_InF_BC31 | GGTAGTCACGATGAGCACGTAGTGATGTATGTGTTTATGGAATAACTAGC |
| Frag1_InF_BC32 | GGTAGGACTGAGATCATGATCGTGATGTATGTGTTTATGGAATAACTAGC |
| Frag1_InF_BC33 | GGTAGACGACATGATACTGCTGTGATGTATGTGTTTATGGAATAACTAGC |
| Frag1_InF_BC34 | GGTAGATACAGCACAGATGTGGTGATGTATGTGTTTATGGAATAACTAGC |
| Frag1_InF_BC35 | GGTAGACAGTCGATATCTCTCGTGATGTATGTGTTTATGGAATAACTAGC |
| Frag1_InF_BC36 | GGTAGGCTCGATCACATGACGGTGATGTATGTGTTTATGGAATAACTAGC |
| Frag1_InF_BC37 | GGTAGGTCGTACACGTGCGACGTGATGTATGTGTTTATGGAATAACTAGC |
| Frag1_InF_BC38 | GGTAGACTCATATCTAGAGTGGTGATGTATGTGTTTATGGAATAACTAGC |
| Frag1_InF_BC39 | GGTAGACTGATCTGTCGCGCTGTGATGTATGTGTTTATGGAATAACTAGC |
| Frag1_InF_BC40 | GGTAGCACTAGCTCTGACTACGTGATGTATGTGTTTATGGAATAACTAGC |
| Frag1_InF_BC41 | GGTAGGCTGTCATGTACTAGCGTGATGTATGTGTTTATGGAATAACTAGC |
| Frag1_InF_BC42 | GGTAGTATACATACACGCACTGTGATGTATGTGTTTATGGAATAACTAGC |
| Frag1_InF_BC43 | GGTAGTGTGACGACGCGTCTCGTGATGTATGTGTTTATGGAATAACTAGC |
| Frag1_InF_BC44 | GGTAGGACGTGAGCATGCACTGTGATGTATGTGTTTATGGAATAACTAGC |
| Frag1_InF_BC45 | GGTAGCTCGATACGTGTAGCTGTGATGTATGTGTTTATGGAATAACTAGC |
| Frag1_InF_BC46 | GGTAGGTGTCTAGACAGCTGTGTGATGTATGTGTTTATGGAATAACTAGC |
| Frag1_InF_BC47 | GGTAGGATGCATGCGTACGCAGTGATGTATGTGTTTATGGAATAACTAGC |
| Frag1_InF_BC48 | GGTAGTATCAGAGCAGCGATGGTGATGTATGTGTTTATGGAATAACTAGC |
| Frag1_InR_BC5 | CCATCTACAGCGACGTCATCGGACATAATTGTTGCCGTCTTGGG |
| Frag1_InR_BC6 | CCATCGCGCAGACTACGTGTGGACATAATTGTTGCCGTCTTGGG |
| Frag1_InR_BC7 | CCATCGTCTCTGCGATACAGCGACATAATTGTTGCCGTCTTGGG |
| Frag1_InR_BC8 | CCATCAGTATGAGATAGCTCGGACATAATTGTTGCCGTCTTGGG |
| Frag1_InR_BC9 | CCATCGCGACGAGTACTCATGGACATAATTGTTGCCGTCTTGGG |
| Frag1_InR_BC10 | CCATCAGTATCACAGTCGCTGGACATAATTGTTGCCGTCTTGGG |
| Frag1_InR_BC11 | CCATCATCATATGATGCGACAGACATAATTGTTGCCGTCTTGGG |
| Frag1_InR_BC12 | CCATCAGACGTAGATCACAGCGACATAATTGTTGCCGTCTTGGG |
| Frag1_InR_BC13 | CCATCCGTGTCATGCTACTCAGACATAATTGTTGCCGTCTTGGG |
| Frag1_InR_BC14 | CCATCTGTGAGACTGCATGTCGACATAATTGTTGCCGTCTTGGG |
| Frag1_InR_BC15 | CCATCGCTCAGTGCGCTACTGGACATAATTGTTGCCGTCTTGGG |
| Frag1_InR_BC16 | CCATCACTATCGCGCACGCAGGACATAATTGTTGCCGTCTTGGG |
| Frag1_InR_BC17 | CCATCTGACACTCTGCACGCGGACATAATTGTTGCCGTCTTGGG |
| Frag1_InR_BC18 | CCATCCAGACGTGACTGATATGACATAATTGTTGCCGTCTTGGG |
| Frag1_InR_BC19 | CCATCGCACTGTAGTGATCGTGACATAATTGTTGCCGTCTTGGG |
| Frag1_InR_BC20 | CCATCCAGTGCGAGACAGTAGGACATAATTGTTGCCGTCTTGGG |
| Frag1_InR_BC21 | CCATCAGTAGTGCTACTCGACGACATAATTGTTGCCGTCTTGGG |
| Frag1_InR_BC22 | CCATCATGCGAGATCTGCTCAGACATAATTGTTGCCGTCTTGGG |
| Frag1_InR_BC23 | CCATCTGAGACATACTGAGTGGACATAATTGTTGCCGTCTTGGG |
| Frag1_InR_BC24 | CCATCATGTGCACTAGTGTACGACATAATTGTTGCCGTCTTGGG |
| Frag1_InR_BC25 | CCATCTCAGCTGACGATGTGAGACATAATTGTTGCCGTCTTGGG |
| Frag1_InR_BC26 | CCATCACTGATGCGCACATGTGACATAATTGTTGCCGTCTTGGG |
| Frag1_InR_BC27 | CCATCCTACTCTCAGCAGTGAGACATAATTGTTGCCGTCTTGGG |
| Frag1_InR_BC28 | CCATCATCTACATCACGACTCGACATAATTGTTGCCGTCTTGGG |
| Frag1_InR_BC29 | CCATCATATAGTACAGCGTCTGACATAATTGTTGCCGTCTTGGG |
| Frag1_InR_BC30 | CCATCGACACGACTAGATCGCGACATAATTGTTGCCGTCTTGGG |
| Frag1_InR_BC31 | CCATCTACGAGTCTGTCATACGACATAATTGTTGCCGTCTTGGG |
| Frag1_InR_BC32 | CCATCACTCAGCTACATAGTGGACATAATTGTTGCCGTCTTGGG |
| Frag1_InR_BC33 | CCATCACGTATCATAGTGAGAGACATAATTGTTGCCGTCTTGGG |
| Frag1_InR_BC34 | CCATCGAGTCGTATCGCTCATGACATAATTGTTGCCGTCTTGGG |
| Frag1_InR_BC35 | CCATCGCGATCACGAGTAGACGACATAATTGTTGCCGTCTTGGG |
| Frag1_InR_BC36 | CCATCCTAGACGTACATGTCGGACATAATTGTTGCCGTCTTGGG |
| Frag1_InR_BC37 | CCATCTAGCAGTCACTGTGCGGACATAATTGTTGCCGTCTTGGG |
| Frag1_InR_BC38 | CCATCCGTCATGCGATAGCTAGACATAATTGTTGCCGTCTTGGG |
| Frag1_InR_BC39 | CCATCGCGCAGTCGTCTGTATGACATAATTGTTGCCGTCTTGGG |
| Frag1_InR_BC40 | CCATCATGAGCTACGTACAGAGACATAATTGTTGCCGTCTTGGG |
| Frag1_InR_BC41 | CCATCGTCGCGAGTCTATCAGGACATAATTGTTGCCGTCTTGGG |
| Frag1_InR_BC42 | CCATCACATCGATCTGCACTAGACATAATTGTTGCCGTCTTGGG |
| Frag1_InR_BC43 | CCATCAGTATAGCATAGACGCGACATAATTGTTGCCGTCTTGGG |
| Frag1_InR_BC44 | CCATCGTGAGAGCGTGACTCTGACATAATTGTTGCCGTCTTGGG |
| Frag1_InR_BC45 | CCATCTGTCAGTAGATGACTCGACATAATTGTTGCCGTCTTGGG |
| Frag1_InR_BC46 | CCATCTCGTACGAGATCGACAGACATAATTGTTGCCGTCTTGGG |
| Frag1_InR_BC47 | CCATCCTACATGTGACTCGAGGACATAATTGTTGCCGTCTTGGG |
| Frag1_InR_BC48 | CCATCGCGCTATAGTGCTCGTGACATAATTGTTGCCGTCTTGGG |

Table S2: Primary PCR conditions

| Volume | Reagents | Cycling Steps | Time |
| --- | --- | --- | --- |
| 17 | H2O | 1. 94ºC | 2 min |
| 2.5 | Buffer | 2. 96ºC | 20 sec |
| 4 | dNTPs | 3. 60ºC | 5 min |
| 0.125 | uL Forw. Primer (10uM) | 4. Repeat to step 2, 34X |  |
| 0.125 | uL Rev. Primer (10uM) | 5. 72ºC | 10 min |
| 0.25 | LA Taq |  |  |
| 1 | uL DNA ("x" pg/uL) | 6. 4ºC | hold |
| 25 | uL Total Reaction Volume |  |  |

Expected size of product from primary PCR is ~5kb

Table S3: Secondary PCR conditions

| Volume | Reagents per reaction | Cycling Steps | Time |
| --- | --- | --- | --- |
| 29.50 | H2O | 1. 94ºC | 2 min |
| 5 | Buffer | 2. 96ºC | 20 sec |
| 8 | dNTPs | 3. 60ºC | 5 min |
| 2.5 | uL Forw. Primer (1uM) | 4. Repeat to step 2, 14X |  |
| 2.5 | uL Rev. Primer (1uM) | 5. 72ºC | 10 min |
| 0.5 | LA Taq |  |  |
| 2 | uL DNA ("x" pg/uL) | 6. 4ºC | hold |
| 50 | uL Total Reaction Volume |  |  |

Expected size of product from the secondary PCR is 5364 base pairs.

Table S4: Genomic context of PacBio sequencing errors

| Lab strains | PacBio  Re-sequenced | Position  ref | Ref^1^  base call | PacBio  base call | Position  PacBio | Context ref | | Context PacBio |
| --- | --- | --- | --- | --- | --- | --- | --- | --- |
| PFL0030c | 3D7_pacbio30_NumReads82 | 1797 | A | **del** | 1796 | | AAAAAAACACA | AAAAA*ACACA |
| PFL0030c | 3D7_pacbio30_NumReads82 | 4822 | C | **del** | 4820 | | TCCCCCCAAGA | TCCCC*CAAGA |
| PFL0030c | 3D7_pacbio70_NumReads119 | 3575 | A | **del** | 3574 | | AAAAAAATGTA | AAAAA*ATGTA |
| PFL0030c | 3D7_pacbio70_NumReads119 | 3982 | A | **del** | 3980 | | AAAAAAACAAT | AAAAA*ACAAT |
| PFL0030c | 3D7_pacbio70_NumReads119 | 4493 | A | **del** | 4490 | | AAAAAAACAAG | AAAAA*ACAAG |
| HB3var2csaA | HB3_pacbio100_NumReads101 | 2841 | A | **del** | 2840 | | AAAAAAACTCC | AAAAA*ACTCC |
| HB3var2csaA | HB3_pacbio30_NumReads167 | 2841 | A | **del** | 2840 | | AAAAAAACTCC | AAAAA*ACTCC |
| HB3var2csaB | HB3_pacbio70_NumReads59 | 1909 | A | **del** | 1908 | | AAAAAAATGAT | AAAAA*ATGAT |
| HB3var2csaB | HB3_pacbio70_NumReads59 | 4571 | A | **del** | 4569 | | TAAAAAATATA | TAAAA*ATATA |
| HB3var2csaB | HB3_pacbio70_NumReads59 | 4813 | A | **del** | 4810 | | AAAAAAATAAT | AAAAA*ATAAT |
| HB3var2csaB | HB3_pacbio30_NumReads70 | 209 | A | **del** | 208 | | AAAAAAAGAAC | AAAAA*AGAAC |
| HB3var2csaB | HB3_pacbio30_NumReads70 | 1909 | A | **del** | 1907 | | AAAAAAATGAT | AAAAA*ATGAT |
| HB3var2csaB | HB3_pacbio30_NumReads70 | 4813 | A | **del** | 4810 | | AAAAAAATAAT | AAAAA*ATAAT |
| HB3var2csaA | HB3_pacbio70_NumReads46 | 10 | A | **del** | 9 | | ATAGTAAAACC | ATAGT*AAACC |
| HB3var2csaA | HB3_pacbio70_NumReads46 | 295 | C | **del** | 293 | | GTGTCCCTCCG | GTGTC*CTCCG |
| HB3var2csaA | HB3_pacbio70_NumReads46 | 2417 | A | **del** | 2414 | | AAAAAAATGTA | AAAAA*ATGTA |
| HB3var2csaA | HB3_pacbio70_NumReads46 | 2841 | A | **del** | 2837 | | AAAAAAACTCC | AAAAA*ACTCC |
| HB3var2csaA | HB3_pacbio70_NumReads46 | 3360 | A | **del** | 3355 | | AAAAAAATTAC | AAAAA*ATTAC |
| HB3var2csaA | HB3_pacbio70_NumReads46 | 3445 | A | **del** | 3439 | | CTCATAAAAAA | CTCAT*AAAAA |
| HB3var2csaA | HB3_pacbio70_NumReads46 | 4052 | A | **del** | 4045 | | AGGAAAACAAA | AGGAA*ACAAA |
| HB3var2csaA | HB3_pacbio70_NumReads46 | 4408 | C | **del** | 4400 | | CCCCCCCAACA | CCCCC*CAACA |
| HB3var2csaA | HB3_pacbio70_NumReads46 | 4625 | A | **del** | 4616 | | TTCTGAAAAAA | TTCTG*AAAAA |
| HB3var2csaB | HB3_pacbio_100_NumReads100 | 4182 | A | **del** | 4181 | | AAAAAAATTAT | AAAAA*ATTAT |
| Ref: reference laboratory strains | | | | | | | | |
