# Supplementary material for: A new method for sequencing the hypervariable Plasmodium falciparum gene var2csa from clinical samples
Source: Malar J. 2017 Aug 17;16:343. doi: 10.1186/s12936-017-1976-8 (PMC5561619; doi:10.1186/s12936-017-1976-8)
Supplement: Supplementary file 2 — Additional file 2: Supplemental figures. [file 12936_2017_1976_MOESM2_ESM.docx]

**A new method for sequencing the hypervariable *Plasmodium falciparum* gene *var2csa* from clinical samples.**

Antoine Dara, Mark A. Travassos, Matthew Adams, Sarah Schaffer DeRoo, Elliott F. Drábek, Sonia Agrawal, Miriam K. Laufer, Christopher V. Plowe, Joana C. Silva

**Additional file 2**


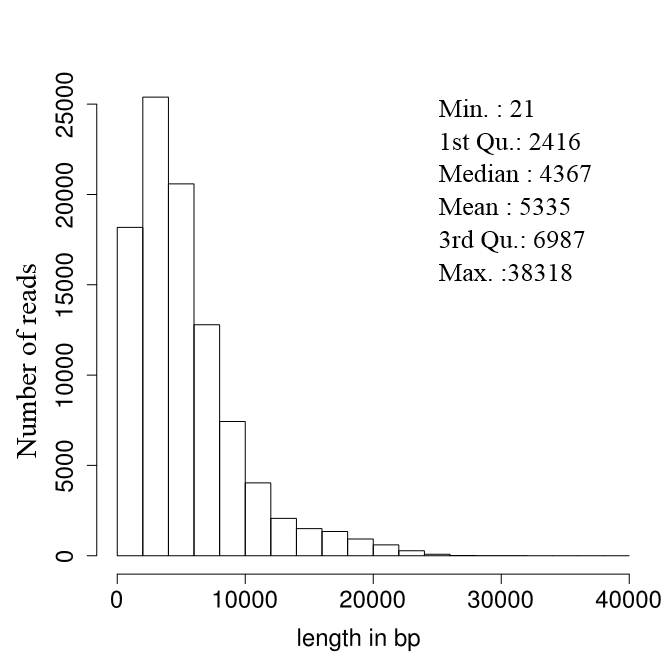
**Figure S1:** Distribution of raw reads or polymerase reads. The number of raw reads is plotted against the length in base pairs. The median and mean read length was 4367 bp, slightly shorter than the amplicon suggesting that most the raw reads had less than one pass.


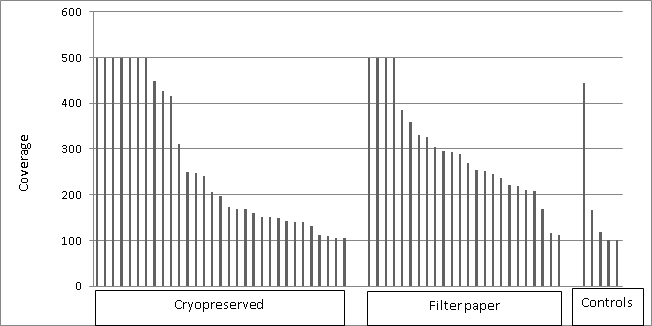


**Figure S2:** Average depth of coverage of consensus sequences. The x-axis represents type of specimen, and the y-axis is the depth coverage. The resequenced 3D7 allele amplified alone, denoted as 3D7_pacbio100, has 445-fold depth coverage; the 3D7_pacbio70 recovered from the 3D7:HB3 mixture (70%:30%, respectively) has 119x depth coverage. All samples have a minimum of 100X coverage. “Controls” were from the laboratory strains 3D7 and HB3.


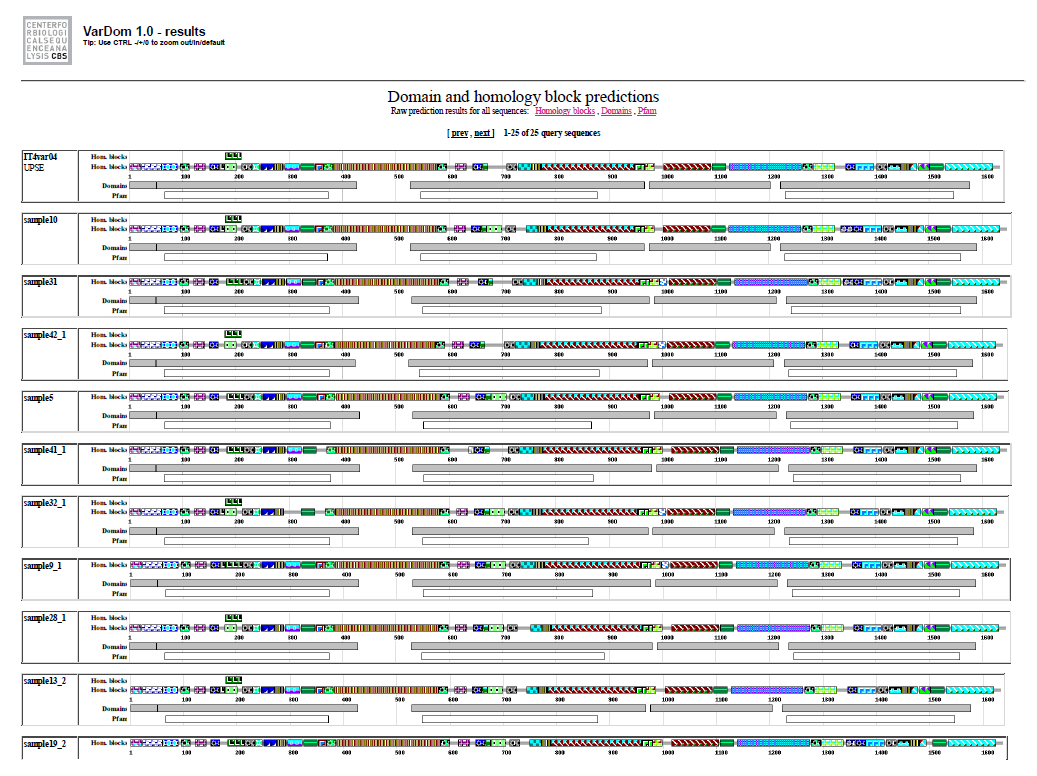
 **Figure S3:** Annotation of VAR2CSA domains using the VarDom server. Laboratory strain *var2csa* IT4var04 and clinical sample (reference VAR2CSA) domains including NTS, DBLpam1, DBLpam2, CIDRpam, and DBLpam3 are respectively represented in grey bars. Above the domains, homology blocks are drawn in colored rectangles. Below these domains, the protein family corresponding to PfEMP1 family is shown in the white rectangle.
